# The presentation of a short adapted questionnaire to measure asthma knowledge of parents

**DOI:** 10.1186/s12887-018-0991-4

**Published:** 2018-01-26

**Authors:** Maaike M. A. Franken, Monique T. M. Veenstra–van Schie, Yasmine I. Ahmad, Hendrik M. Koopman, Florens G. A. Versteegh

**Affiliations:** 1 Groene Hart Ziekenhuis, Department of Pediatrics, POBox 1098, 2800 BB Gouda, the Netherlands; 2 Faculty of Medicine, Leiden University, Leiden, the Netherlands; 3 Faculty of Clinical Psychology, Leiden University, Leiden, the Netherlands; 4 Department of Pediatrics, Ghent University Hospital, Ghent, Belgium; 5 Beatrix Children’s Hospital, University Medical Centre Groningen, Groningen, the Netherlands

**Keywords:** Asthma, Asthma knowledge, Questionnaire, Dutch, Validation, Parents, Children

## Abstract

**Background:**

The aim of this study is to establish asthma knowledge of parents of children (0–18 years) with asthma at the outpatient clinic.

**Methods:**

A translated and adapted a 21 item Likert type 5 point scale questionnaire (Cronbach’s α-coefficient 0.73) was completed by 291 parents of children with asthma. Total asthma knowledge scores were associated with demographic and psychosocial variables.

**Results:**

Factor analysis resulted in a new reduced 10 item questionnaire (Cronbach’s α-coefficient 0.72). Higher educational level of parents was associated with better asthma knowledge (*p* < 0.008 and *p* < 0.003). Parents showed more knowledge (*p* < 0.001) on non-medication questions. Asthma knowledge of the parent did not correlate with child age, gender, duration of airway problems, time since diagnosis or severity of asthma.

**Conclusions:**

Education of parents concerning the working mechanism, indications and use of asthma medications are an essential part of asthma education. Asthma education should be repeated frequently to parents of children with long-term airway problems or diagnosed asthma. Special attention must be paid to parents with only high school education or less.

## What is known-what is new

Asthma knowledge is important for both patients and caregivers. Most questionnaires to evaluate this knowledge are limited, outdated and/or only for adults. We adapted and evaluated a new questionnaire for the caregivers of asthmatic children. This 10-item-containing questionnaire proved to be reliable and may be used to establish the asthma knowledge of parents in a short period of time. It showed the importance to adjust asthma education to the educational level of the parents and to pay extra attention to (the use of) medication.

## Background

Asthma is the most common chronic disease in childhood and affects an estimated 300 million individuals worldwide [1, 2]. International and national guidelines have therefore produced recommendations for effective asthma management based on the best scientific information available [3].

The Global Initiative for Asthma (GINA) states that an important part of effective asthma management is to give adults and children with asthma the ability to control their own condition with guidance from health care professionals. Guided self-management reduces asthma morbidity and anxiety in children [4]. Adult education significantly reduced future hospital admissions and improved symptom control [5]. Education is one of the six essential features to achieve guided self-management [1], including the importance of a basic understanding of asthma pathophysiology for children and their parents, as part of an effective pediatric asthma treatment [6].

Increased knowledge of asthma was associated with improved lung function and self efficacy, a reduction of restricted activity days, school absenteeism, visits to an emergency department, and fewer nights disturbed by asthma [7].

Education for caregivers of children with asthma resulted in a significant increase in asthma knowledge, management behaviour and quality of life [8]. The introduction of educational programs on asthma knowledge calls for instruments to measure its effectiveness [8–20]. In the Netherlands there is one questionnaire available for adults with asthma. But the questions focus on asthma medication [16]. In order to assess the knowledge of parents of children with asthma in the Netherlands, we used an already validated international asthma knowledge questionnaire [14]. The aim of this study is to evaluate the value of this questionnaire to establish asthma knowledge of parents of children (0–18 years) with asthma.

## Methods

### Questionnaire

The study has a cross-sectional design. The questionnaire (Table 1) is a translated version of the asthma knowledge Likert type 5 point scale questionnaire for parents of children with asthma, containing 17 questions, as developed by Rodriguez-Martinez [14]. Because of the lack of medication specific questions (reliever medication vs. preventive medication) 4 questions from an other asthma knowledge questionnaire were added [11]. The first asthma knowledge questionnaire [14] is chosen because of its adequate Chronbach’s α coefficient of 0.73 for their study population and the multidimensional concept of asthma [14]. Although Pink et al. in a review was unable to identify any high-quality patient-centered asthma knowledge outcome measures [21], we decided that this asthma knowledge questionnaire was the best option for our study.

**Table 1:** 21 item Asthma Knowledge Questionnaire: factor analysis and reliability

Items	Factor I^b^	Factor II^b^
1. Inhaler use can lead to dependence or addiction.^a^	X	
2. Inhalers can have an affect on the heart or damage it.^a^	X	
3. It’s not good for children to use the inhaler for too long.^a^	X	
4. After a child’s asthma attack, once the coughing is over, use of the inhaler and medications should stop.		
5. Children with asthma should use asthma medications only when they have symptoms (coughing, congestion, or wheezing).^a^	X	
6. It’s better to use inhalers directly, without a holding chamber, so the medication can go more directly to the lungs.^a^	X	
7. The main cause of asthma is airway inflammation.		
8. Parents should ask a doctor to tell the school that an asthmatic child shouldn’t exercise or participate in physical education classes.^a^		X
9. Children who have asthma shouldn’t participate in sports that make them run too much.^a^		X
10. When a child has an asthma attack it’s best to go to the emergency room even if symptoms are mild.^a^	X	
11. Asthma attacks can be prevented if medications are taken even when there are no symptoms—between attacks.		
12. Flu infections are the main causes or triggers of asthma attacks.		
13. It’s best not to smoke or let anyone else smoke near a child who has asthma.		
14. If the parents of a child with asthma smoke outside the house, it won’t affect the child.		
15. If an asthmatic child gets the flu, you should apply the inhalers even if there’s no coughing or wheezing.		
16. Asthmatic children might have attacks that are severe enough to require hospitalization in an intensive care unit or they might even die from an attack.		
17. Some medications for asthma don’t work unless they’re administered every day.^a^	X	
18. With preventer medications, it does not matter if some doses are missed or if you go on and off them.^a^	X	
19. You should use ‘preventer medication’ when you have an asthma attack.		
20. Parents should give ‘reliever medication’ to a child as soon as they recognize the first sign of asthma.		
21. Blue puffer (Ventolin), Brown puffer (Flixotide), and Green puffer (Serevent) are called ‘preventer medications’, so they should be used everyday although you are well.		

^a^Questions of the short form 10-item-questionnaire

^b^Factor I indicates the use and working mechanism of inhalators and asthma medication with an individual Cronbach’s α coefficient of 0.67. Factor II indicates sports and asthma with an individual Cronbach’s α coefficient of 0.75

The questions were translated from English to Dutch in a systematic way with the forward backward method [22]. The translation was done by two independent native Dutch speakers; then a consensus translation was formed from these two translations by three of the authors. Subsequently a third independent native Dutch speaker back-translated the Dutch translation into English and compared it with the original questionnaires. A few minor adjustments were made and a pilot study (containing 7 parents of children with asthma) was performed. Questions were clarified when the meaning appeared unclear.

A Likert-type scale of 5 points was used to respond to each of the 21 questions. The items were graded ‘1’, ‘2’, ‘3’, ‘4’, and ‘5’ for ‘strongly disagree’, ‘disagree’, ‘neither agree, nor disagree’, ‘agree’, and ‘strongly agree’, respectively. Negative items were reverse coded by subtracting the responses values from 5. Missing answers of the asthma knowledge questionnaire scored a ‘3’. Total score was calculated by the sum of score item responses, ranging from 21 to 105, with higher scores indicating greater knowledge of asthma.

This implies that the correct response is always ‘strongly agree’ for all questions.

An additional form was added to establish parental educational level (highest achieved level by mother and father), day of birth and sex of the child, severity of asthma according to the parents (question: rate the severity from 0 (none) till 10 (very severe)), the date of the child’s first appointment (which concerned airway problems) at our hospital and the date of diagnosis. The date of the first appointment and the date of diagnosis were used to estimate the duration of time parents have been aware of their children’s airway problems and/or their diagnosis of asthma.

### Patients

Subjects were recruited from the database of the GOUDA (**G**eneral **O**utcome **U**sing HRQoL- **D**iagnostic measures in children with **A**sthma) study, a longitudinal study about health related quality of life in children with asthma or bronchial hyper reactivity (0–18 years) conducted in the Groene Hart Ziekenhuis, a general teaching hospital in Gouda, the Netherlands. Inclusion criteria for the Gouda Study were: all children with doctor diagnosed reversible bronchial hyper reactivity or asthma, diagnosed before the age of 15 years. Excluded were children with concomitant disease, like heart disease, diabetes mellitus or mental disability, insufficient understanding of the Dutch language, as estimated by the researchers.

Parents of all participants of the GOUDA study received, after informed consent, a questionnaire, in total 536 questionnaires were sent out. Families with two or more children received only one questionnaire for one child.

### Statistical analysis

Statistical Package for the Social Sciences (SPSS for Windows and Mac, version 20) was used to analyse all data. Internal consistency of the questionnaire was analysed by the Chronbach’s α coefficient [23]. Factor analysis was used to investigate construct validity. Correlations levels between total asthma knowledge scores and continuous variables were assessed by Pearson’s correlation coefficients. A one way analysis of variance (ANOVA) was used to examine the association of total asthma knowledge scores with categorical variables. Comparisons groups were made by Student T-tests. A significance level of 0.05 was used for all analyses.

## Results

Table 2 shows the characteristics of the children and their parents. The response rate was 54.3% (291 out of 536 questionnaires) and 92.6% of the questionnaires were filled in by the mother of the child.

**Table 2:** Patients characteristics

Variable	No. of Patients, %
Age, years	
0–5	74 (26.2)
6–12	124 (43.8)
13–18	85 (30.0)
Sex	
Male	173 (61.1)
Female	110 (38.9)
Mother’s educational level	
No education	1 (0.4)
Primary and secondary school	5 (1.8)
High school	42 (14.8)
Professional education	110 (38.9)
Bachelor degree	97 (34.3)
Master degree	27 (9.5)
Father’s educational level	
No education	3 (1.1)
Primary and secondary school	7 (2.5)
High school	54 (19.1)
Professional education	103 (36.4)
Bachelor degree	63 (22.3)
Master degree	50 (17.7)
Native country responder	
The Netherlands	266 (94.0)
Morocco	10 (3.5)
Other than above	7 (2.5)
	Mean (SD)
Duration airway problems in our hospital (years)	5.0 (4.36)
Time since diagnosis (years)	5.42 (4.54)
Severity of asthma according to the parents (0–10)	4.28 (1.97)

A total of 8 questionnaires were excluded (2.7%) for various reasons: substantial part not completed, already completed for another member of the family, not being a participant of the GOUDA study or the participant added a column ‘I don’t know’ to the questionnaire. From the 283 patients that were included, in a small number there were missing data for which the parents were contacted by telephone. Complete the data or these data were added by the research team according to the data in the patient’s personal record. Missing data on time since diagnosis or other answers (with a maximum of 3 missing answers) were added by the research team through interpolation.

The mean age of the children with asthma was 9.7 years (StD dev 4.8 y), median age 10.4 years, (61.1% males). This percentage is in accordance with the common population were the prevalence of asthma in boys is nearly twice as great as in girl before the age of 14 [1].

By means of factor analysis and varimax rotation a smaller questionnaire with a Cronbach’s α coefficient of 0.72 was established containing 10 questions (Table 1). Two factors were identified by factor analysis with an individual Cronbach’s α coefficient of 0.67 and 0.75 (Table 1). The first factor included questions related to the use and working mechanism of inhalators and asthma medication, the second factor included questions related to sports and asthma. This 10-item-containing questionnaire is a short and reliable test, which may be used to establish the asthma knowledge of parents in a short period of time.

### Asthma knowledge scores

The mean parental asthma knowledge score in this study is 76.8 (StD dev 5.97, range 6093). The median score is 77.

According to the parents asthma knowledge was not associated with child age, child gender, duration of airway problems, time since diagnosis or severity of asthma.

One way ANOVA showed a significant difference between parental educational level groups (*p* < 0.015). A higher educational level of both parents was associated with higher asthma knowledge. Post-hoc test’s showed that mothers with a bachelor degree scored significantly higher (*p* < 0.008) than mothers with a ‘high school’ or lower as highest completed educational level (Table 3). Fathers with a master degree scored significantly higher (*p* < 0.003) than fathers with a ‘primary and secondary school’ or ‘high school’ as the highest completed educational level.

**Table 3:** One way ANOVA total asthma knowledge score in relation to the mother’s educational level

Mother’s educational level	Mean (SD)
High school	73.4 (5.2)
Professional education	76.0 (6.0)
Bachelor degree	77.1 (5.2)*
Master degree	76.0 (7.2)

* *p* < 0.008

To investigate the (joint) effect of educational level of father and mother on total knowledge score a multiple regression analysis was run with educational levels as the predictor and total score as criterium. Result shows that the full model with the two predictors was significant (R2 = .07, F(2, 276) = 9.70, *p* < .001). This finding was mainly because of the effect of father’s educational level. For each increase of one unit on educational level the total score is expected to be .95 units higher. This effect was significant (b = .95, SEb = .36, *p* = .009). Parents who were born in the Netherlands had higher asthma knowledge scores then parents born abroad with significantly higher scores than parents born in Morocco (*p* < 0.001. Parents scored significantly higher (*p* < 0.001) on non-medication questions in comparison to questions concerning asthma medication.

## Discussion

The mean parental asthma knowledge score of this study, 76.8%, is quite similar to previous results [14, 24].

In our study increased knowledge was not associated with time since diagnosis. One might expect that parents who had more years of experience with asthma management and more contact with health care providers, would as a result receive more education about asthma, and subsequently would have more asthma knowledge. Our results suggest that asthma education however should regularly be repeated even to parents of children with long-term airway problems or diagnosed asthma [19, 20].

Parental educational level was (consistent with previous reports) a significant predictor of asthma knowledge [15, 25]. One study showed that lower educated mothers often had poorer knowledge about asthma medication use [25]. In our study higher education was associated with greater asthma knowledge. Our results give an indication that, as long as parents continue their education past high school, they might show a higher level of asthma knowledge. This is confirmed in another study, in which children with persistent asthma whose parents had education beyond high school were more likely to use inhaled corticosteroids daily than those whose parents had less education [26]. Therefore, special care and time for asthma education of parents with a high school education or less is advocated.

That parents with a higher level of education show better knowledge about asthma might be expected as they are more likely to have prior knowledge by their previous education and therefore more easily absorb new information.

Interestingly, parents in our study scored significantly lower on questions concerning asthma medication in comparison to non-medication questions. This finding suggests that more attention should be paid to the education of parents concerning the working mechanism, indications and use of asthma medications. The addition of the new items on medication to the questionnaire makes it difficult to compare this study with the overall findings in the original study.

The finding that parents born in the Netherlands scored significantly higher than parents born abroad should be interpreted with caution because of the small sample size of the parents born abroad (6%).

The Chronbach’s α coefficient of the original questionnaire was 0.73 when used in a Spanish speaking population [14]. One other study used the questionnaire to evaluate an asthma medication training program for immigrant Mexican community health workers [24]. A second study identified factors associated with recurrent emergency department visits for asthma exacerbations in children in Bogotá, Colombia [27]. The Cronbach’s α coefficient of our questionnaire, containing 21 questions, was 0.48. When including only the original 17 questions [14], the Cronbach’s α coefficient was also low, (0.41). Other statistical analyses showed comparable results.

When looking at the discrepancy of the two Cronbach’s α a few possible causes should be considered. As described in the results we constructed, by means of factor analysis and varimax rotation, a smaller questionnaire of 10 questions with a Cronbach’s α coefficient of 0.72 (Table 1). The content validity was accurate and the translation process into Dutch precise. The original questionnaire was designed for a Spanish population and therefore the wording of the questions might not be suitable for other populations. The low statistical reliability might be related to the fact that the asthma knowledge level of our Dutch study population is different from South America. However, this contradicts our results that show a mean parental asthma knowledge score of 76.8% for the questionnaire. A possible explanation is that parents who did not know the answer to a question filled out ‘neither agree, nor disagree’, scoring 3 points. In this way a parent without asthma knowledge could have received an asthma knowledge score of 63 points, consistent with 60% of the questionnaire. Another possibility is that questionnaires should be developed per cultural entity since different populations cope in different ways with disease [28, 29].

There are some limitations to our study. In the first place we did not record precise clinical data on the patients who were invited but did not participate, but they did not differ in age, sex or ethnicity. Another limitation is that we did not record whether one or both parents had asthma themselves, which might influence their knowledge about asthma.

The differences with the outcome from the studies in Spanish speaking populations might indicate that social and cultural differences influence the usefulness of questionnaires, as we have shown before in asthma [28] and HIV [30] and that questionnaires possibly have to be adapted for different populations.

Another limitation is that we did not separate questions to parents of preschool children from questions to parents of older subjects but this was comparable with the original questionnaire from Rodriquez-Martinez [14].

As was indicated the development and description of the asthma knowledge questionnaire did not fully comply with the requirements of a psychometrically sound instrument. So our results should be interpreted with some caution [21].

Therefore the results of our study may not be generalised. In future studies these items should be analysed as well as the importance of the time interval between the last visit and the completion of the questionnaire.

## Conclusions

The results of our study suggest a need to improve the asthma education of parents of children with asthma in general. Special attention should be paid to parents with only high school education or less. Education of parents concerning the working mechanism, indications and use of asthma medications are an essential part of asthma education [31]. Not only should parents of children recently diagnosed with asthma receive asthma education (written action plans included), but also parents of children with long-term diagnosed asthma should receive recurrent updates.

A reliable 10-item-containing questionnaire is designed, which may be used to establish the asthma knowledge of parents in a short period of time. Further research is needed to explore the usefulness in different countries and populations.

## Abbreviations

ANOVA: Analysis of variance
GINA: Global Initiative for Asthma
GOUDA: **G**eneral **O**utcome **U**sing HRQoL- **D**iagnostic measures in children with **A**sthma
HRQoL: Health-related quality of life
SPSS: Statistical Package for the Social Sciences
